# Stem Cell-Based Cell Therapy for Glomerulonephritis

**DOI:** 10.1155/2014/124730

**Published:** 2014-06-09

**Authors:** Meiling Jin, Yuansheng Xie, Qinggang Li, Xiangmei Chen

**Affiliations:** ^1^Department of Nephrology, Chinese PLA General Hospital, Chinese PLA Institute of Nephrology, State Key Laboratory of Kidney Diseases, National Clinical Research Center of Kidney Diseases, 28 Fuxing Road, Beijing 100853, China; ^2^Medical College, Nankai University, Tianjin 300071, China

## Abstract

Glomerulonephritis (GN), characterized by immune-mediated inflammatory changes in the glomerular, is a common cause of end stage renal disease. Therapeutic options for glomerulonephritis applicable to all cases mainly include symptomatic treatment and strategies to delay progression. In the attempt to yield innovative interventions fostering the limited capability of regeneration of renal tissue after injury and the uncontrolled pathological process by current treatments, stem cell-based therapy has emerged as novel therapy for its ability to inhibit inflammation and promote regeneration. Many basic and clinical studies have been performed that support the ability of various stem cell populations to ameliorate glomerular injury and improve renal function. However, there is a long way before putting stem cell-based therapy into clinical practice. In the present article, we aim to review works performed with respect to the use of stem cell of different origins in GN, and to discuss the potential mechanism of therapeutic effect and the challenges for clinical application of stem cells.

## 1. Introduction

The glomerulonephritis (GN) is a heterogeneous group of clinical conditions that are generally, but not always, characterized by inflammation of the glomeruli with secondary tubulointerstitial and vascular changes. They may be primary disorders or a secondary manifestation of systemic diseases and are believed to share an immune-mediated pathogenesis [1, 2]. GN is a common cause of end stage renal disease (ESRD) worldwide especially in developing countries such as China and India [3]. GN is a major contributor to the escalating health burden associated with chronic kidney disease. Thus, broader implementation of interventions shown to be effective in slowing the progression of GN is very important from an economic perspective [4, 5].

Therapeutic options for glomerulonephritis applicable to all cases mainly include symptomatic treatment and strategies to delay progression. Regular clinical follow-up [6], blood-pressure control [7], and the use of an inhibitor of angiotensin-converting enzyme [8, 9] are proven to be beneficial to therapeutic measures. Traditional immunosuppressive therapies for GN include corticosteroids and cytotoxic agents, which have been used since the 1950s [2]. Corticosteroids are effective in several types of glomerulonephritis owing to their ability to inhibit activity of the transcription factor nuclear factor **κ**B and consequently inhibit the proinflammatory effects of cytokines known to promote glomerular inflammation actively, including interleukin 1*β* (IL-1*β*) and tumour necrosis factor **α** (TNF-**α**). However, other actions of corticosteroids result in well recognized side effects that limit the usefulness of these drugs. Currently used immunosuppressive therapies for GN are not uniformly effective and are frequently associated with serious side effects [5]. Studies in animal models and patients have attempted to find new treatment, which could regulate inflammation, reduce the injury, contribute to the renal regeneration, and is less toxic.

Stem cells (SCs) are undifferentiated cells characterized by their ability for self-renewal and the capacity for multilineage differentiation [10]. Stem cell-based therapies have the potential effectivity in many human diseases including kidney disease, and it have been proven to be safe and effective in a wide range of immunomediated diseases [11, 12]. However, besides haematopoietic stem cell (HSC) transplantation for the treatment of haematological disorders and some dermal and corneal indications, all other approaches based on stem cell remained experimental medical research, while the desperation of patients who find no hope for the cure of their diseases would choose the stem cell-based therapies [13]. The emerging field of regenerative medicine is progressing rapidly and is supported by many studies including animal experiment and preclinical experiment. Stem cell-based therapy may have the potential to be developed into the novel therapeutic approach to GN.

The first study of stem cell in experimental GN was published in 1999; Imasawa et al. performed bone marrow transplantation (BMT) on a murine model IgA nephropathy and investigated that BMT from normal donors could attenuate glomerular lesions [14]. During the past 14 years, many preclinical and clinical studies have been performed that support the ability of various stem cell populations to have the therapeutical effect on GN. The purpose of this paper is to critically review the works performed with respect to the use of stem/progenitor cells in GN, both at the experimental and clinical levels, and to discuss the mechanism underlying the beneficial effect of SCs and the possible translation in therapeutic approaches for clinical application of GN.

## 2. Stem Cell Types Investigated Heretofore in Glomerulonephritis

Stem cells are undifferentiated, self-renewing cells that possess a multilineage differentiation potential. In mammals, stem cells could be classified as embryonic stem cells (ESCs), fetal and adult stem cells (ASCs), and induced pluripotent stem cells (iPSCs) [15, 16]. The preclinical and clinical studies that have assessed the use of stem cell in GN are summarized in Tables 1 and 2, respectively. In these studies, we broadly separated stem cells used for treatment on GN into two categories: bone marrow-derived stem cells and nonbone marrow stem cells.

### 2.1. Bone Marrow-Derived Stem Cells

Bone marrow-derived cells (BMDCs) are a heterogeneous population which is consisting of mesenchymal stem cells (MSCs), hematopoietic stem cells (HSCs), and endothelial progenitors (EPCs); they have the capacity to differentiate into cells of endothelia, epithelial, cardiomyocyte, and neuronal lineage [47, 48]. As the relatively greater concentration of stem cells in the bone marrow and the ease of procurement of these cells, bone marrow-derived stem cells have been used in most of the basic, preclinical, and clinical studies in GN.

#### 2.1.1. Mesenchymal Stem Cells (MSCs)

MSCs, also known as marrow stromal cells or multipotential stromal cells, typically express CD73, CD105, CD90, and STRO-1 but lack hematopoietic markers (CD45, CD34, and CD14/CD11b) [49]. MSCs represent a class of adult progenitor cells capable of differentiation to a variety of cell types, including chondrocytes (cartilage cells), osteoblasts (bone cells), adipocytes (fat cells), and more lineages (myogenic, cardiomyogenic, etc.). Due to this ability, confirmed by the results of either in vitro or vivo studies, MSCs became an attractive tool in the context of tissue engineering and cell-based therapy [50]. Indeed, the ease of handing and enormous expansion potential of MSCs, together with the low immunogenicity, provide a nearly unlimited number of cells suitable for a wide range of syngeneic and allogeneic therapeutic applications [51]. MSCs are attractive candidates for renal repair, because nephrons are of mesenchymal origin and the stromal cells are of crucial importance for signaling, leading to differentiation between both nephrons and collecting ducts [52]. In addition, MSC has been shown to possess immunomodulatory capabilities [53, 54], so it would be efficacious in the glomerulopathy treatment.

MSCs often refer to bone marrow mesenchymal stem cells (BM-MSCs), but MSCs also include umbilical cord MSCs (UC-MSCs), adipose-derived MSCs (AD-MSCs), and fetal membranes MSCs (FM-MSCs), which would be discussed later. BM-MSCs are a rare population of fibroblast-like cells resident in the stroma of the bone marrow and represent about 0.001–0.01% of the nucleated cells [51]. As the most commonly used type of SCs, the results of BM-MSC administration in animal models and patients with GN have been encouraging.

#### 2.1.2. Hematopoietic Stem Cells (HSCs)

HSCs reside in the bone marrow and differentiate into cells of both myeloid (monocytes and macrophages, neutrophils, basophils, eosinophils, erythrocytes, megakaryocytes/platelets, and dendritic cells) and lymphoid lineages (T-cells, B-cells, and NK-cells) [55]. Since the 1990s, autologous hematopoietic stem cells transplantation (HSCT) has been proven to be a choice for patients with systemic lupus erythematosus (SLE) or other refractory autoimmune diseases. In 1996, the first international registry of stem cell transplantation classified SLE as one indication for autologous hematopoietic stem cell transplantation [45]. Although its therapeutical effect on lupus nephritis has been reported, its effects on any other GN need further investigation.

#### 2.1.3. Endothelial Progenitors (EPCs)

Endothelial progenitors (EPCs) are mobilized into peripheral blood in response to certain cytokines and/or tissue ischemia and homing to the site of neovascularization, hormone-induced ovulation, corneal neovascularization, wound healing, and cerebral ischemia by differentiating into endothelial cells (reendothelialization) [56–58]. CD34 is a typical surface marker of both HSCs and EPCs [59, 60]. CD34^+^ cells have been found in the bone marrow and peripheral blood and have the potential to give rise to all blood cell types as well as endothelial cells [61], which have potential for glomeruli repair.

### 2.2. Nonbone Marrow Stem Cells

#### 2.2.1. Adipose-Derived Mesenchymal Stem Cells (AD-MSCs)

Bone marrow-derived MSC is the most used MSC in cell-based therapy and tissue engineering. However, the use of BM-MSCs still has some limitations because of the low number of MSCs found in bone marrow aspirates and the invasive procedure when obtaining them. Compared with BM-MSCs, adipose-derived MSCs (AD-MSCs) are equally capable of differentiating into cells and tissues of mesodermal origin. In addition, they offer some advantages as an attractive, readily available adult stem cell because of the ease of harvest and their abundance [36].

#### 2.2.2. Umbilical Cord Mesenchymal Stem Cells (UC-MSCs)

UC-MSCs could be isolated from human umbilical cord without harming the donor and are free of ethical problems [62]. Compared with BM-MSCs, UC-MSCs represent an abundant source for large quantity production [40].

#### 2.2.3. Fetal Membranes Mesenchymal Stem Cells (FM-MSCs)

Human fetal tissues have been found to be rich sources of mesenchymal stem cell, and several studies have shown that MSC could be isolated from human fetal membranes (FM) [63, 64]. Fetal membranes are generally discarded as medical waste after delivery, and there are no ethical concerns associated with the use.

## 3. Therapeutical Effect of Stem Cells on Glomerulonephritis

### 3.1. Mesangial Proliferative Glomerulonephritis (mesPGN) and IgA Nephropathy

Anti-Thy 1.1 glomerulonephritis is a classic model of mesangioproliferative glomerulonephritis characterized by mesangiolysis followed by repair via mesangial cell proliferation, mesangial matrix accumulation, and monocyte/macrophage influx [65]. Many studies have shown that BM-MSC treatment could improve the renal function and histological changes, by reducing mononuclear infiltration, attenuating mesangial activation and glomerular endocapillary proliferation [18, 19, 52]. Tsuda et al. have reported that in the anti-Thy 1 nephritis rats FM-MSCs could significantly reduce activated mesangial cell proliferation, glomerular monocyte/macrophage infiltration, mesangial matrix accumulation, and the glomerular expression of inflammatory or extracellular matrix-related genes including TNF-*α*, monocyte chemotactic protein-1 (MCP-1), type I collagen, TGF-*β*, and type 1 plasminogen activator inhibitor (PAI-1) [22]. In rat anti-Thy 1.1 glomerulonephritis, EPCs also have been reported to participate in glomerular endothelial and mesangial cell turnover and contribute to microvascular repair [20, 21, 66]. In another experimental model of mesPGN, Abe-Yoshio et al. injected habu snake venom (HSV) or saline intravenously after endothelial progenitor cells transplantation on mice and found that labeled EPCs were increased in damaged glomeruli and vascular endothelial cell growth factor; vascular endothelial growth factor (VEGF) overexpression was detected in glomerular epithelial and endothelial cells, mesangial cells, and EPCs, suggesting EPCs participation in glomerular capillary repair of damaged glomeruli in HSV-induced glomerulonephritis [23].

In 1999, Imasawa et al. [14] performed bone marrow transplantation (BMT) on a murine model IgA nephropathy and investigated that BMT from normal donors could attenuate glomerular lesions. Three years later, he reported remission of IgA nephropathy after allogeneic stem cell transplantation in a 28-year-old patient [24].

### 3.2. Lupus Nephritis (LN)

Ma et al. reported that allogeneic BM-MSCs transplantation could ameliorate nephritis in lupus mice via inhibition of B-cell activation [67]. In recent years, it has been reported that UC-MSCs could meliorate clinical parameters and histology by improving kidney fibrosis and modulating the inflammatory response (including inhibiting lymphocytes, inducing polarization of Th2 cytokines, and inhibiting proinflammatory cytokines) in experimental lupus nephritis [27, 28]. Good et al. found that hematopoietic stem cell transplantation could be used to prevent and cure lupus-like diseases in mice (BXSB mice and W/BF1 mice) [26]. In 1996, the first international registry of stem cell transplantation classified SLE as one indication for autologous hematopoietic stem cell transplantation [45]; HSCs transplantation could ameliorate lupus nephritis (LN) in the patients with SLE [33, 43, 45, 46]. El-Ansary et al. [29] performed MSC transplantation on 10 patients with SLE, and then they showed a greater decline of their serum creatinine and elevation of mean creatinine clearance levels after MSC injection compared with the control group.

### 3.3. Diabetic Nephropathy (DN)

In the diabetic animal model induced by streptozotocin (STZ), BM-MSCs could ameliorate diabetic nephropathy via blood sugar reduction and/or renal protective actions of the MSCs within the glomeruli via inhibiting macrophage infiltration, protecting podocyte, and promoting healing [30–32, 34, 68, 69]. In another study, Chen et al. reported that infusion of BMDCs obtained from *db*/*m* donors to *db*/*db* recipient mice benefited microvascular function, insulin sensitivity, and nephropathy [35]. Fang et al. have reported that autologous transplantation of AD-MSCs could ameliorate STZ-induced diabetic nephropathy in rats by inhibiting oxidative stress, proinflammatory cytokines, and the p38 MAPK signaling pathway [36]. In addition, Masoad et al. investigated that mononuclear cells treatment was superior to pioglitazone in controlling hyperglycemia, improving the renal structure and function changes, and reducing renal laminin expression associated with STZ-induced diabetic nephropathy in rats [37].

### 3.4. Focal Segmental Glomerulosclerosis (FSGS)

In experimental FSGS (Adriamycin-induced nephropathy rats), BM-MSCs limited podocyte loss and apoptosis and partially preserved nephrin and CD2AP. BM-MSCs attenuated the formation of glomerular podocyte-parietal epithelial cell bridges and normalized the distribution of NCAM^+^ progenitor cells along the Bowman's capsule, thereby reducing glomerulosclerosis [38, 39]. In another study, UC-MSCs could attenuate the progression of FSGS by improving kidney fibrosis and modulating the inflammatory response [40]. In the clinical studies, Belingheri et al. found that after the allogeneic bone marrow mesenchymal stem cells infusions, the patient with focal segmental glomerulosclerosis (FSGS) had a stable renal function and the proteinuria target was reached without plasmapheresis and some circulating inflammatory factors decreased and were still low after one year [44].

### 3.5. Antiglomerular Basement Membrane Glomerulonephritis

Suzuki et al. have reported therapeutic effects of human mesenchymal stem cells in Wistar-Kyoto rats with antiglomerular basement membrane glomerulonephritis. Five days after nephrotoxic serum nephritis was induced, Wistar-Kyoto rats were given human MSCs (3 × 10^6^); the results showed that hMSC-treated rats had decreased kidney weight, proteinuria, and glomerular tuft area; the serum creatinine level and degree of glomerular crescent formation were decreased by hMSC treatment. In addition, ED-1-positive macrophages, CD8-positive cells, and TUNEL-positive apoptotic cells in glomeruli were reduced. Renal cortical mRNA for TNF-*α*, IL-1*β*, and IL-7 and the serum IL-17A level were decreased, whereas renal cortical mRNA for IL-4 and Foxp3 and the serum IL-10 level were increased. It is demonstrated that anti-inflammatory and immunomodulatory effects were involved in the mechanism of attenuating established experimental anti-GBM GN by hMSCs [42].

### 3.6. Antineutrophil Cytoplasmic Antibody (ANCA)-Associated Renal Vasculitis (AAV)

Gregorini et al. have reported the first case of MSCs treatment in a patient with severe renal ANCA-associated vasculitis in whom standard therapy was not feasible. After two times of MSCs infusion (1.5 × 10^6^ cells/kg body weight), this patient remained in clinical remission without any therapy in 12 months after the second MSC infusion [70].

## 4. Potential Mechanisms of Actions of Stem Cells in Glomerulonephritis

**Figure 1:**
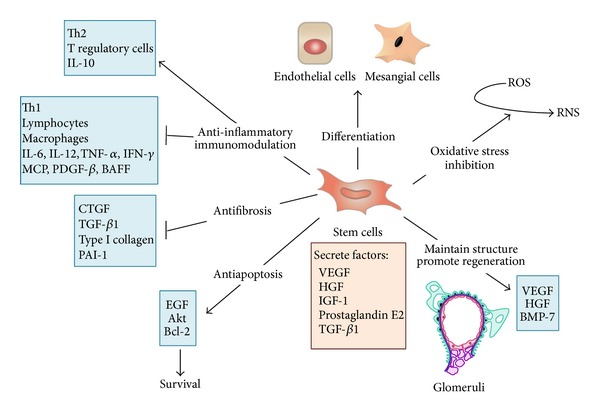
Potential mechanisms of therapeutical effect of stem cells on glomerulonephritis (GN). Stem cell-based cell therapy is likely to improve the kidney injury in GN mainly via (trans)differentiation of transplant cells into renal cells and paracrine mechanisms. Paracrine mechanisms include anti-inflammatory and immunomodulatory, antifibrosis, antiapoptosis, oxidative stress inhibition, and maintaining normal structure and regeneration. (IL: interleukin, TNF: tumor necrosis factor, IFN-*γ*: interferon *γ*, MCP: monocyte chemotactic protein, PDGF-*β*: platelet-derived growth factor *β*, BAFF: B-cell activating factor, CTGF: connective tissue growth factor, TGF-*β*: transforming growth factor-*β*, PAI-1: plasminogen activator inhibitor 1, EGF: epithelial growth factor, VEGF: vascular endothelial growth factor, HGF: hepatocyte growth factor, IGF-1: insulin-like growth factor, BMP-7: bone morphogenic protein-7, ROS: reactive oxygen species, RNS: reactive nitrogen species.).

Taken together, the studies reviewed above suggest that stem cell-based cell therapy is likely to improve the kidney injury in GN. However, the mechanism responsible for the beneficial effects of stem cell remains largely unknown. Currently, the mechanisms of the therapeutic effect of stem cell on GN could be mainly summarized as (trans)differentiation of transplant cells into renal cells and paracrine mechanisms (Figure 1). However, most of the evidences showed that the paracrine mechanisms play a leading role in glomerular repair.

### 4.1. (Trans)Differentiation of Transplant Cells into Renal Cells

Dispute remains over whether the transplant cells could (trans)differentiate into renal cells to promote tissue repair. Rookmaaker et al. found that bone marrow-derived endothelial cells contribute to the glomerular endothelial cell and mesangial cell turnover in pathologic conditions [66]. It has been reported that bone marrow transplant-derived podocytes were found in wild-type mice and genetic mouse models with diffuse mesangial sclerosis in a frequency of approximately 10% at 13 weeks after transplantation. However, several recent studies have suggested that stem cells especially MSCs may mediate their beneficial effects through paracrine activity, not through a differentiation mechanism as had been described in some acute renal damage models as well as GN models [71]. Meyer-Schwesinger et al. have investigated that podocyte replacement by bone marrow-derived cells after initial podocyte injury or as contribution to a continuous normal turnover did not occur in the puromycin aminoglycoside and renal ablation models in rats [41]. Kunter et al. also reported that MSCs accelerated glomerular recovery from mesangiolytic damage which is possibly not related to differentiation into resident glomerular cell types in experimental glomerulonephritis, because 85 to 95% of transplanted MSC that localized in glomeruli failed to express endothelial, mesangial, or monocyte/macrophage markers even through renal artery injection [52]. These evidences suggest that the direct contribution of transplanted cells to tissue regeneration is minimal. Currently, this discrepant result could not be definitely explained but may be due to different GN types and/or different species and/or different stem cell.

### 4.2. Paracrine Mechanisms

It was reported that MSCs would express several growth factors such as hepatocyte growth factor (HGF), vascular endothelial growth factor (VEGF), and insulin-like growth factor-1, all known to improve renal function, mediated by their antiapoptotic, mitogenic, and other cytokine actions. These paracrine actions of MSCs result in the renal downregulation of proinflammatory cytokines interleukin 1 (IL-1), tumor necrosis factor *α* (TNF-*α*), interferon *γ* (IFN-*γ*), and inducible nitric oxide synthase and upregulation of anti-inflammatory and organ-protective interleukin-10, as well as basic fibroblast growth factor, TGF-*β*, and antiapoptotic Bcl-2 [19]. All of these effects contribute to the glomerular repair.

#### 4.2.1. Anti-Inflammatory and Immunomodulatory

MSCs have strong immunosuppressive activity. It modulated the immune response mainly by suppressing T-cell proliferation, influencing DC maturation and function, suppressing B-cell proliferation and terminal differentiation, modulating monocytes/macrophages infiltration and function, and inhibiting proinflammatory cytokines production [52]. The underlying mechanism for stem cell might relate to differential T_H_1/

T_H_2 cytokine profiles. Previous studies have shown that upregulation of Th1 cytokine IL-2 and IFN-*γ* together with a decreased production of Th2 cytokine IL-4 might upregulates autoantibody produced by B-cells and is associated with disease activity [72–74]. In both of experimental lupus nephritis and focal segmental glomerulosclerosis, administration of UC-MSCs increases IL-4 and IL-10 and decreases IL-2 and IFN-*γ*, suggesting that UC-MSCs delay autoimmunity by modulating T-cell differentiation and shift Th1 to Th2 polarization [28, 40]. MSCs may work through secreting transforming growth factor-*β* (TGF-*β*), indoleamines 2,3-dioxygenase, and prostaglandin E_2_, thereby promoting Th1 to Th2 polarization and anti-inflammatory dendritic cell type 2 signaling [75]. In lupus mice, transplantation of BM-MSCs downregulates the expression and secretion of IFN-*γ* and upregulates the levels of TGF-*β*, leading to reduced levels of serum and peripheral B-cell activating factor (BAFF) expression. Reduction of BAFF downregulates T1, T2, and mature B-cell population and then plays a role in disease progression. In addition to the prominent inhibitory effects on T-cells, MSCT may modulate B-cell populations through its effects on BAFF [25]. Many studies have shown that the beneficial effect of SCs might be mediated by downregulation of proinflammatory cytokines (IL-6, IL-12, TNF*α*, IFN-*γ*) and upregulated anti-inflammatory cytokines (IL-10) [32, 40]. In addition, UC-MSCs treatment also inhibited expression of high-mobility group box 1 (HMGB-1), which could stimulate an inflammatory cytokine response [27]. Macrophage is a key inflammatory cell mediating kidney inflammation. Activated macrophages could mediate renal injury by elaborating a series of proinflammatory, antiangiogenic, and profibrotic factors such as IL-1*β*, IL-6, and TNF*α* [76]. Monocyte chemotactic protein-1 (MCP-1) is mainly responsible for recruiting and activating monocytes that promote macrophage accumulation and activation. MCP-1 expression level is significantly increased in GN process and correlate with the number of infiltrating macrophages [77]. MSCs treatment could inhibit expression of MCP-1 through a prostaglandin E_2_-depentdent mechanism [22] or HGF via disrupting nuclear factor-kappa B signaling pathway [32, 78, 79]. In experimental glomerulonephritis, monocytes were found to invade the glomerulus and cause glomerular injury by releasing ROS (reactive oxygen species) and inflammatory cytokine [80]. The activated monocytes which infiltrate the glomerulus express Ron and are recruited into tuft by the chemotactic effect of MSP (macrophage-stimulating protein) [81, 82]. In addition, TGF-*β* and PDGF-*β* are also chemoattractants for monocytes, which are released in glomerulus in anti-Thy 1 disease. It is found that MSCs could suppress glomerular MSP and Ron expression and decrease the local level of platelet-derived growth factor *β* (PDGF-*β*) and circulating level of TGF-*β*. This effect of reduction of infiltrating monocytes promotes the attenuation of glomerular injury [18].

#### 4.2.2. Antifibrosis

Many mediators are involved in the development of scarring and fibrotic conditions in renal pathological process. MSC therapies were tested in lung, heart, and chronic diseases and were found to be effective on reducing the fibrosis [83]. TGF-*β*1 is known to play an important role in the pathogenesis of glomerulosclerosis and tubulointerstitial fibrosis in progressive renal disease [84, 85]. Connective tissue growth factor (CTGF) expression has been found to be lower when there was no obvious interstitial disease, while it has been found to be increasing as the disease became worse. TGF-*β*1 could enhance the activity of CTGF-induced fibroblasts [86]. Ma et al. have found an increase in TGF-*β*1 and CTGF expression in the kidneys of ADR-treated rats that could be prevented by UC-MSC transplantation [40]. In addition, FM-MSCs were reported to reduce the glomerular expression of several genes involved in fibrogenesis including type I collagen, TGF-*β*, and PAI-1 [22].

#### 4.2.3. Antiapoptosis

In Adriamycin-induced nephropathy, the regenerative effect of MSC-derived VEGF on ADR-damaged podocytes was explored in coculture setting. In agreement with the results in vivo data, Zoja et al. found that MSCs significantly enhanced viability and limited apoptosis of podocytes in response to a toxic concentration of ADR. Such a prosurvival effect was markedly abrogated by a neutralizing anti-VEGF antibody, thereby indicating a cytoprotective action of VEGF on podocytes. Many studies have shown that MSC-induced VEGF induced the activation of Akt, a key factor in the regulation of prosurvival signals, in several cells types, thus reducing apoptosis [39, 87, 88]. In vitro, Li and colleagues have found that human AD-MSCs protect podocytes from apoptosis induced by high glucose via secretion of epithelial growth factor (EGF) [89].

#### 4.2.4. Inhibition of Oxidative Stress

In studies of MSCs treatment on diabetic nephropathy, it was shown that MSCs could ameliorate GN by inhibiting oxidative stress [36]. MSCs are resistant to ex vivo culture and ionizing radiation, two conditions that generate a strong oxidative stress [90]. It is reported that the low susceptibility of MSCs to the deleterious effect of ROS and reactive nitrogen species correlates with the ability of these cells to effectively scavenge peroxide and peroxynitrite, due to the constitutive expression of SOD1, SOD2, CAT, and GPX1 enzymes and high levels of glutathione. MSCs possess the main enzymatic mechanisms to detoxify reactive species and to prevent oxidative damage of the proteome and genome [91].

#### 4.2.5. Maintaining Normal Structure and Regeneration

In experimental glomerulonephritis of anti-Thy 1 nephritis, excessive mesangial proliferation is activated by growth factors that include MSP, PDGF-*β*, and IL-6. Activated proliferating mesangial cells express *α*-SMA and proliferating cell nuclear antigen (PCNA). MSCs translation could reduce the number of PCNA-positive cells in the mesangial cell proliferating phase and maintained steady growth. Such stabilization was associated with levels of PDGF-*β*, MSP, and IL-6 lower than in untreated rats. Rampino et al. suggested that MSCs regulate the repair response driven by PDGF-*β*, MSP, and IL-6, making glomerular proliferation less aggressive and more steady [18]. In ADR nephropathy, Zoja et al. showed that repeated MSC injections by exerting a remarkable antiapoptotic effect limited podocyte depletion and partially restored nephrin and CD2AP expression. The protective effect of MSCs against podocyte dysfunction and loss translated into less number of adhesions between parietal epithelial cells (PECs) and podocytes, with the reestablishment of a normal localization of neural cell adhesion molecule (NCAM-)positive PECs along the Bowman's capsule. Thus, stem cell therapy by reducing podocyte injury restored parietal progenitor cell regenerative capacity thereby ameliorating glomerular architecture and preventing sclerotic lesions. VEGF, highly produced in vitro and vivo by MSCs, may play an important role in regulating and maintaining podocyte and glomerular endothelial cell integrity and function, contributing to limit migration and proliferation of PECs of the Bowman's capsule thereby reducing the early formation of PEC-podocyte bridges. This inhibition of PEC-podocyte activation is also related to the anti-inflammatory effects of MSCs. VEGF may exert a beneficial effect on podocytes; this is supported also by data showing that nephrin and CD2AP, target proteins regulated by VEGF, were partially preserved in renal tissue of MSC-treated animals [39].

In anti-Thy 1 disease, the lysis of the mesangial stalk causes loss of glomerular capillaries, rarefaction of the capillary network and formation of microaneurysms [18]. The reconstruction of new glomerular capillaries is necessary for wound healing. The HGF/Met system play a key role in the neoangiogenesis, exogenous administration of HGF in anti-Thy1 disease has been shown to contribute to glomerular repair though promoting proliferation of endothelial cells and regeneration of glomerular capillaries [92]. Rampino et al. found that treatment with MSC induced a striking expression of the Met receptor in glomerular capillaries, suggesting that MSC-induced Met expression made local HGF operative and that the HGF/Met system mediated the reconstruction of glomerular capillaries [18]. It is noteworthy that MSCs release high amounts of proangiogenic VEGF and profibrotic TGF-*β* and thereby exert beneficial effects in the recovery process in experimental glomerulonephritis, and this effect was also investigated in bone marrow-derived angiogenic cells [20]. Apart from paracrine effects of MSC, it also is possible that intrarenally injected MSC served as chemoattractants and “feeders” to circulation of hematopoietic stem cells and thereby further promoted resolution of injury [52]. Endothelial progenitor cells (EPCs) also augment tissue neovascularization and contribute to reendothelialization after endothelial injury by secreting VEGF in habu-snake venom-induced glomerulonephritis [23]. However, Wang et al. reported that MSCs ameliorate podocyte injury in diabetic nephropathy rat model via secreting higher levels of BMP-7 (bone morphogenic protein-7) but not VEGF. BMP-7 is a podocyte survival factor, important for the maintenance of podocyte viability and differentiation [34]. It is noteworthy that in the FM-MSC treatment on anti-Thy 1 disease, there was no significant induction of VEGF or HGF expression in the kidney seen after FM-MSC transplantation. Therefore, contribution of FM-MSC-derived growth factors might be minimal in the repair process of anti-Thy nephritis [22].

Given evidences that beneficial effects of stem cells are mostly paracrine, some investigators have tested whether the administration of a cell-free “cocktail” of factors secreted by SCS, that is, cell culture supernatants, might be equally effective as the whole SCs [93, 94]. However, in the process of glomerular injury resolution, a mix of several cytokines, if probably required, suggests that cytokines therapy using a single protein or gene could not work. It is noteworthy that effects of cytokines may be conflicting in different studies, such as TGF-*β*. TGF-*β* is a prototypical hypertrophic and fibrogenic cytokine [95]. It has been suggested to play a key role in the pathogenesis of tubulointerstitial fibrosis and glomerulosclerosis in progressive renal disease. It also could cause glomerular basement membrane (GBM) thickening and may promote podocyte apoptosis or detachment [96, 97]. However, MSC releases high amounts of profibrotic TGF-*β*, which could promote Th1 to Th2 polarization and accelerate glomerular recovery from mesangiolytic damage [52, 75]. The balance between beneficial and harmful effects of cytokines seems to depend on the cytokine microenvironment [98].

## 5. Potential Limitations to Clinical Translation

Among the studies above, there are a few ones evaluating the therapeutical effect on patients with primary or secondary GN. As SLE has been one indication for hematopoietic stem cell transplantation, the majority of the clinical studies are about the HSC transplantation on LN. The rest are all case reports, in which the enrolled patient who found no hope for the cure of his disease would choose the stem cell therapy [44, 70, 99]. Despite the encouraging results from various types of SCs therapies on animal model studies, no cell type has been demonstrated to be effective and appropriate in alleviating GN in patients. Thus, before jumping into clinical practice, many questions need to be solved, such as the optimal cell type, the optimal cell dose, the optimal frequency of treatment, and the optimal route of cell administration. Although human biology is only partially predictable from experimental animal models, preclinical studies remain an important element in the scientific development of stem cell-based therapy, which provide fundamental basis for clinical practice. Although there is no obvious side effect investigated in animal experiments, the potential risks of SC therapy on human should be considered. Unwanted side effects include the potentially proangiogenic role in tumor formation and adoption of unwanted phenotypes (maldifferentiation), which need to be studied more systematically in the future [17, 100]. The past 50 years of medicine have shown treatment advances in the field of hematopoietic stem cells transplantation (HSCT); however, kidney complications of HSCT attract physicians in recent years. It is noteworthy that glomerular diseases after hematopoietic stem cell transplantation are increasing; membranous glomerulonephritis and minimal change disease are the most forms of glomerular diseases noted in patients with graft versus host disease after hematopoietic stem cell transplantation [101]. The common causes of kidney diseases include HSCT-associated thrombotic microangiopathy, calcineurin inhibitor nephrotoxicity, and chronic graft-versus-host disease-associated glomerulonephritis [102]. As the risk of thrombosis and embolization exists and may worsen the process of GN, the kidney complications of stem cell-based therapy should be evaluated before clinical practice.

## 6. Conclusion

Stem cell-based therapy appears to be a safe treatment modality in patients with GN. Although the majority of studies in the field of stem cell-based therapy and GN shows remarkable benefits; they are mostly confined to small animal models and the clinical studies are limited to case reports. However, never has a novel therapy translated from preclinical models to humans so quickly. Before putting stem cell therapy into use in clinical practice, what we should do and what is important are (1) to resolve issues concerning optimal cell type, dosage, timing, and route of administration and (2) to proceed with rigorous, rationally designed, large-scale, randomized clinical trials. We believe that stem cell-based therapy is likely to become a clinical reality that revolutionizes the management of GN.

## Figures and Tables

**Table 1:** Animal studies of stem cell therapy in glomerulonephritis (GN).

Authors (year)	Animal model (host)	Type of GN	Type of stem cell	Time of cell therapy	Dose and route of administration	Follow-up period after cell therapy	Outcomes	Reference number
Kunter et al. (2007)	Anti-Thy1,1 nephritis (Wistar/Lewis rats)	mesPGN	BM-MSCs	2 d after disease induction	2 × 10^6 ^ cells left renal artery injection	10 d	↑recovery from mesangiolysis ↑glomerular cell proliferation ↓proteinuria	[17]

Rampino et al. (2011)	Anti-Thy1,1 nephritis (Sprague-Dawley rats)	mesPGN	BM-MSCs	3 d after anti-Thy1 injection	3 × 10^6 ^ cells tail vein injection	11 d	↑renal function ↓glomerular monocyte influx, ↓renal injury	[18]

Sakr et al. (2013)	Anti-Thy1,1 nephritis (albino rats)	mesPGN	BM-MSCs	5 d after anti-Thy1 injection	1 × 10^6 ^ cells tail vein injection	28 d	↓renal injury ↓apoptosis, *α*-SMA ↑renal function	[19]

Uchimura et al. (2005)	Anti-Thy1,1 nephritis (rats)	mesPGN	BMDCs	5 wk before anti-Thy1 antibody injection	5 × 10^7^ cells tail vein injection	28 d	↑microvascular repair ↑differentiation into glomerular endothelial and mesangial cell	[20]

Li et al. (2006)	Anti-Thy1,1 nephritis (rats)	mesPGN	BMDCs	5 wk before experimental progressive glomerulosclerosis	1-2 × 10^8 ^ cells tail vein injection	12 wk	↑renal function ↑improvement in glomerular hemodynamics ↓renal injury	[21]

Uchimura et al. (2005)	Anti-Thy1,1 nephritis (Lewis rats)	mesPGN	BM-EPCs	1 d after antibody injection	1.0 ± 0.2 × 10^6^ cells left renal artery injection	6 d	↓glomerular injury score, the area positive for mesangial *α*-SMA, infiltration of macrophages	[20]

Tsuda et al. (2010)	Anti-Thy1,1 nephritis (Lewis rats)	mesPGN	FM-MSCs	2 d after anti-Thy1 antibody injection	5 × 10^5 ^ cells tail vein injection	14 d	↓urinary protein excretion, activated mesangial cell, glomerular monocyte/macrophage infiltration, mesangial matrix accumulation, TNF-*α*, MCP-1, type I collagen, TGF-*β*, PAI-1	[22]

Abe-Yoshio et al. (2008)	Habu-snake venom-induced glomerulonephritis (TIE2/L mice)	mesPGN	BM-EPCs	On the same date of disease induced	1 × 10^6^ cells tail vein injection	56 d	↓renal injury	[23]

Imasawa et al. (1999)	HIGA mice (high content of serum IgA)	IgA nephropathy	BMDCs		1 × 10^7^ cells intravenously injection	26 wk	↓serum IgA ↓renal injury	[14]

Imasawa and Utsunomiya (2002)	High serum level IgA ddY mice	IgA nephropathy	BM-MSCs	6 h after Gy injection	10 × 10^6 ^ cells	26 wk	↓mesangial recipients of IgA and C3, glomerular sclerosis, IgA level	[24]

Ma et al. (2012)	MRL/lpr mice	LN	BM-MSCs		1 × 10^6 ^ cells intravenous injection	26 wk	↓BAFF, IL-10 ↑TGF-*β*	[25]

Good et al. (2002)	BXSB mice and (NZW × BXSB) F1 W/BF1 mice	LN	Bone marrow cells HSC		15 × 10^6^ cells	16 wk or 30 wk	↓renal injury	[26]

Gu et al. (2010)	MRL/lpr mice	LN	UC-MSCs	at the 18th, 19th, and 20th wk of age	1 × 10^6^ cells		↑renal function ↓renal injury ↓MCP-1, HMGB-1	[27]

Chang et al. (2011)	NZB/W F1 mice	LN	UC-MSCs		1 × 10^6^ cells tail vein injection	8 months	↑renal function ↓renal injury ↓TNF-*α*, IL-6, IL-12 ↓IL-4, IL-10	[28]

El-Ansary et al. (2012)	STZ-induced DN (C57BL/6 mice)	DN	BM-MSCs	25 d after the first STZ dose	0.5 × 10^6^ cells tail vein injection	62 d	↑renal function normal histologically glomeruli	[29]

Zhou et al. (2009)	STZ-induced DN (Sprague-Dawley rats)	DN	BM-MSCs	4 wk after STZ	2 × 10^6^ cells left cardiac ventricle	8 wk	↑renal function ↓renal mass index	[30]

Ezquer et al. (2009)	STZ-induced DN (C57BL/6 mice)	DN	BM-MSCs	4 wk after STZ	2 doses of 0.5 × 10^6^ cells tail vein injection	4 months	↓sclerosis, mesangial expansion, tubular dilatation, proteins cylinders, podocytes lost	[31]

Lv et al. (2013)	STZ-induced DN (Wistar rats)	DN	BM-MSCs	8 wk after establishment of diabetes model	2 × 10^6^ tail vein injection	8 wk	↑renal function ↓glomerulosclerosis, MCP-1, IL-1*β*, IL-6, TNF*α*	[32]

Thirabanjasak et al. (2010)	STZ-induced DN (Sprague-Dawley rats)	DN	BM-MSCs	4 wk after diabetes onset	1 × 10^6^ cells kidney-targeted ultrasound-targeted microbubble destruction	8 wk	↓renal damage ↓TGF-*β*1 ↑synaptopodin, IL-10	[33]

Wang et al. (2013)	STZ-induced DN (Sprague-Dawley rats)	DN	BM-MSCs	30 d after diabetes induction by STZ injection	2 × 10^6^ cells renal artery injection	60 d	↑renal function ↓podocyte injury ↑BMP-7	[34]

Chen et al. (2009)	db/db mice	DN	BMDCs		1 × 10^6^ cells tail vein injection	50 d	↑renal function ↓renal injury	[35]

Fang et al. (2012)	STZ-induced DN (Sprague-Dawley rats)	DN	AD-MSCs	4 wk after STZ injection	10 × 10^6^ cells renal artery injection	12 wk	↓renal injury, oxidative damage ↓p38, p-ERK, p-JNK	[36]

Masoad et al. (2012)	STZ-induced DN (rates)	DN	Mononuclear cells		150 × 10^6^ cells/rat tail vein injection	8 wk	↓renal function ↓renal injury	[37]

Magnasco et al. (2008)	ADR-induced nephropathy (Rats)	FSGS	BM-MSCs	Concomitantly to ADR/60 d after ADR	10 × 10^6^ cells tail vein injection	24 h	↓podocytes apoptosis ↓renal injury	[38]

Zoja et al. (2012)	ADR-induced nephropathy (Rats)	FSGS	BM-MSCs	36 h, 60 h, 3 d, 5 d, 7 d, 14 d, and 21 d	2 × 10^6^ cells tail vein injection	30 d	↓podocyte loss, apoptosis ↑preserve nephrin and CD2AP ↑improvement in histological parameters	[39]

Ma et al. (2013)	ADR-induced nephropathy (Sprague-Daoley rats)	FSGS	Human UC-MSCs	1, 8, 15, and 22 d	2 × 10^6^ cells tail vein injection	12 wk	↑improvement in clinical parameters and histology↓IL-6, TNF-*α*, CTGF ↑IL-10	[40]

Meyer-Schwesinger et al. (2011)	Puromycin aminoglycoside and renal ablation models (Wistar rats)	FSGS	BM-EPCs	8 wk before model induced	2 × 10^6^ cells intravenously injection	10 wk	↑renal function ↓renal injury	[41]

Suzuki et al. (2013)	Wistar-Kyoto rats	Anti-GBM GN	Human BM-MSCs	4 d after rats induced	3 × 10^6^ cells intravenous injection	13 d	↑improvement in functional and histological parameters ↓collagen types I and III, TGF-*β* ↓ED1-positive macrophages, CD8-positive cells, and TUNEL-positive apoptotic cells in glomeruli ↓renal cortical mRNA for TNF-*α*, IL-1*β*, IL-17a ↑renal cortical mRNA for TNF-*α*, IL-1*β*, IL-17, and serum IL-17A	[42]

mesPGN—mesangial proliferative glomerulonephritis.

LN—lupus nephritis.

STZ— streptozocin.

DN—Diabetic Nephropathy.

FSGS—Focal Segmental Glomerulosclerosis.

BM-MSCs—bone marrow mesenchymal stem cells.

BMDCs—bone marrow-derived cells.

BM-EPCs—bone marrow endothelial progenitors.

FM-MSCs—fetal membranes mesenchymal stem cells.

UC-MSC—umbilical cord mesenchymal stem cells.

AD-MSCs—adipose-derived mesenchymal stem cells.

*α*-SMA—*α*-smooth muscle actin.

BAFF—B-cell activating factor.

IL—interleukin.

TGF-*β*—transforming growth factor-*β*.

MCP-1—monocyte chemotactic protein-1.

HMGB-1—high-mobility group box 1.

TNF-*α*—tumour necrosis factor *α*.

BMP-7—bone morphogenic protein.

CTGF—connective tissue growth factor.

**Table 2:** Clinical trials of stem cell therapy in glomerulonephritis (GN).

Study/name of the trial	Study design	Number of patients	Type of GN	Cell type	Delivery method	Cell dose	Follow-up period	Outcomes	Side effects in cell-treated patients	Reference number
Jayne et al. (2004)	Nonrandomized, uncontrolled study	53 (33 had nephritis)	LN	Autologous hematopoietic stem cell	Vein infusion		26 (0–78) months	Stabilization or improvement in renal function; no patients developed new renal involvement	No major complications reported	[43]

Thirabanjasak et al. (2010)	Case report	1	LN	Autologous hematopoietic stem cell	Renal injection			No improvement in renal function	Development of angiomyeloproliferative lesions at the sites of infection when 3 month after cell therapy	[33]

El-Ansary et al. (2012)	Nonrandomized, uncontrolled study	10	LN	Mesenchymal stem cell	Infusion intravenously	0.7–1 × 10^6^ cell/kg in two divided doses 1 week apart	6 months	↓Scr ↑creatinine clearance	No major complications reported	[29]

Belingheri et al. (2013)	Case report	1	FSGS	Human allogeneic bone marrow mesenchymal stem cells	Vein infusion	1 × 10^6^ cell/kg	22 months	Normal and stable renal function (Scr↓, GFR↑)	No major complications reported	[44]

Su et al. (2013)	Nonrandomized, uncontrolled study	5 (kidney involved in 4)	LN	Autologous peripheral blood hematopoietic stem cell	Vein infusion		40–83 months	All went into clinical remission in 3–6 months; two recurred at the end of follow-up period	No major complications reported	[45]

Alchi et al. (2013)	Nonrandomized, uncontrolled study	28 (17 had nephritis)	LN	Haematopoietic stem cell transplantation	Infusion intravenously		38 months	↑improvement in renal function	No major complications reported	[46]

Rampino et al. (2011)	Case report	1	Renal ANCA-associated vasculitis	Bone marrow mesenchymal stromal cells	Infusion intravenously	1.5 × 10^6^ cell/kg	12 months	Clinical remission	No major complications reported	[18]

LN—lupus nephritis.

FSGS—focal segmental glomerulosclerosis.
